# Proof of Concept for a Quick and Highly Sensitive On-Site Detection of SARS-CoV-2 by Plasmonic Optical Fibers and Molecularly Imprinted Polymers

**DOI:** 10.3390/s21051681

**Published:** 2021-03-01

**Authors:** Nunzio Cennamo, Girolamo D’Agostino, Chiara Perri, Francesco Arcadio, Guido Chiaretti, Eva Maria Parisio, Giulio Camarlinghi, Chiara Vettori, Francesco Di Marzo, Rosario Cennamo, Giovanni Porto, Luigi Zeni

**Affiliations:** 1 Department of Engineering, University of Campania Luigi Vanvitelli, Via Roma 29, 81031 Aversa, Italy; chiara.perri@unicampania.it (C.P.); francesco.arcadio@unicampania.it (F.A.); luigi.zeni@unicampania.it (L.Z.); 2 Moresense Srl, Filarete Foundation, Viale Ortles 22/4, 20139 Milano, Italy; g.dagostino@moresense.tech (G.D.); g.chiaretti@moresense.tech (G.C.); g.porto@moresense.tech (G.P.); 3 Operative Unit of Chemical-Clinical and Microbiological Analysis, San Luca Hospital, Usl Toscana Nord Ovest, 55100 Lucca, Italy; eva.parisio@uslnordovest.toscana.it (E.M.P.); giulio.camarlinghi@uslnordovest.toscana.it (G.C.); chiara.vettori@uslnordovest.toscana.it (C.V.); 4 UOC Chirurgia Generale, Ospedale Valtiberina, Usl Toscana Sud-Est, 52037 Sansepolcro, Italy; francesco.dimarzo@uslsudest.toscana.it (F.D.M.); rosario.cennamo@uslsudest.toscana.it (R.C.)

**Keywords:** SARS-CoV-2, optical-chemical sensors, molecularly imprinted polymers (MIPs), surface plasmon resonance (SPR), optical fiber sensors

## Abstract

The rapid spread of the Coronavirus Disease 2019 (COVID-19) pandemic, caused by the Severe Acute Respiratory Syndrome Coronavirus 2 (SARS-CoV-2) pathogen has generated a huge international public health emergency. Currently the reference diagnostic technique for virus determination is Reverse Transcription Polymerase Chain Reaction (RT-PCR) real time analysis that requires specialized equipment, reagents and facilities and typically 3–4 h to perform. Thus, the realization of simple, low-cost, small-size, rapid and point-of-care diagnostics tests has become a global priority. In response to the current need for quick, highly sensitive and on-site detection of the SARS-CoV-2 virus in several aqueous solutions, a specific molecularly imprinted polymer (MIP) receptor has been designed, realized, and combined with an optical sensor. More specifically, the proof of concept of a SARS-CoV-2 sensor has been demonstrated by exploiting a plasmonic plastic optical fiber sensor coupled with a novel kind of synthetic MIP nano-layer, especially designed for the specific recognition of Subunit 1 of the SARS-CoV-2 Spike protein. First, we have tested the effectiveness of the developed MIP receptor to bind the Subunit 1 of the SARS-CoV-2 spike protein, then the results of preliminary tests on SARS-CoV-2 virions, performed on samples of nasopharyngeal (NP) swabs in universal transport medium (UTM) and physiological solution (0.9% NaCl), were compared with those obtained with RT-PCR. According to these preliminary results, the sensitivity of the proposed optical-chemical sensor proved to be higher than the RT-PCR one. Furthermore, a relatively fast response time (about 10 min) to the virus was obtained without the use of additional reagents.

## 1. Introduction

The rapid spread of the COVID-19 pandemic has generated a huge international public health emergency [1]. The pathogen responsible for COVID-19, initially called 2019-nCoV and then SARS-CoV-2 [2], is a single positive strand RNA virus belonging to the betacoronavirus genus (Coronaviridae family) and is closely related to other coronaviruses responsible for similar respiratory syndrome (SARS) contagions in 2002-2003 [3] and more recent MERS-CoV (2012).

Like other coronaviruses [4,5,6], the 2019-nCoV exploits a glycosylated spike (S) protein, protruding from the viral surface, to enter the host cell. This surface protein consists of two functional subunits: the S1 subunit, which contains the receptor-binding-domain (RBD) responsible for host cell receptor recognition and binding, and S2 subunit, which is involved in viral and host membrane fusion. It is already known that this SARS-CoV-2 entry into the host cell is mediated by its binding to the host cell receptor ACE2 (Angiotensin-converting enzyme 2) [7,8].

The speed of transmission of this emerging coronavirus has made its containment extremely difficult [9] and has forced national healthcare systems to reduce their daily activities to a minimum [10]. Diagnostic testing becomes a particularly important tool, in the absence of an effective therapy or a vaccine, to improve patient management and potentially save lives by limiting the contagion [11,12]. To this end, detection sensitivity is crucial for early-stage diagnostics.

Currently, the presence of the virus in patients is routinely determined by molecular techniques which identify viral RNA through nucleic acid reverse transcription and amplification, Reverse Transcription Polymerase Chain Reaction (RT-PCR) [13]. However, the above technique is not readily deployable in the field due to the high cost of real-time PCR machines and the expertise needed to carry out the analysis. Furthermore, the huge demand for testing has caused shortages of reagents and materials and serious difficulties in the service provision of laboratories. 

Real-time PCR based diagnosis takes at least 3 h including sample preparation. Moreover, collected samples (oral or nasopharyngeal swabs, sputum, bronchoalveolar lavage fluid etc) are often stored, after sampling, up to 24–48 h prior to analysis. 

Consequently, the SARS-CoV-2 pandemic highlighted the need for more suitable and effective tools for the detection and prevention of outbreaks [14]. In fact, the shortcomings related to the use of oropharyngeal swabs have strongly emerged due to the need for laboratory post analysis and as a result of inaccuracies stemming from data transmission and handling [15]. The adopted approach has proven to be slow in response delivery, onerous in terms of human resources and laden with problems in data transmission which inspired ineffective choices in terms of prevention measures.

Moreover, in pandemic events the use of swabs for laboratory analysis could prove to be a great hindrance in crisis management in that the gap between the high demand and low offer of the required materials cannot easily and rapidly be bridged and therefore creates bottlenecks along the entire material and service supply chain. After this SARS-CoV-2 pandemic, in order to obtain a low-cost, simple to use and small-size sensing platform to detect the virus in the population, novel sensor systems will be required to analyze biological fluids in real-time and transmit the results via the Internet, exploiting an Internet of Things (IoT) approach.

Thus, the realization of simple, low-cost, rapid and point-of-care diagnostics has become a global priority. Many efforts have been done in realizing more rapid diagnostic tools, mostly based on the determination of antibodies in blood [16,17] and few concerning sensing devices for the detection of the virus in other biological samples. Recently, Seo G. et al. realized a graphene-based Field-Effect Transistor (FET) biosensor for detecting SARS-CoV-2 in clinical specimens [18]. The authors used an antibody as the recognition element.

This kind of sensors are interesting because they represent a general-purpose sensing platform, able to monitor a reprogrammable Molecular Recognition Element (MRE), and could be highly desirable to face the next crisis.

Alternatively, surface plasmon resonance sensing platform is technologically suitable to monitor specific receptors (natural or synthetic). In fact, surface plasmon resonance (SPR) is widely exploited as an optical detection method for monitoring interactions between an analyte in solution and an MRE immobilized on the SPR sensor [19].

SPR is based on the interaction of light and free electrons in a semi-transparent noble metal layer placed on a dielectric substrate [20]. When a biological or chemical receptor (ligand) is bonded on the metallic layer surface, its interaction with the target molecules changes the refractive index at the outer interface, and this variation is detected by optical interrogation. The sensitivity of the plasmonic phenomenon exponentially decreases with the distance from the metal-dielectric interface as the effective interaction length is usually no larger than a few hundred nanometers. So, in SPR detection, the MRE coupled with a metal surface (usually gold), selectively recognizes and captures the substance in the sample, producing a local variation of the refractive index. The extent of the change in the refractive index depends on the thickness of the selective layer (for example molecularly imprinted polymers) and on the structure and size of the target element (virus, molecule, etc).

Several SPR sensors have been realized to date, from the classic prism-based configurations to the latest fiber-optic-based ones [21,22,23]. Among the latter, SPR sensors based on plastic optical fibers (POFs) are particularly suitable for the development of very low-cost, simple, and small-size sensor devices due to some advantageous properties of the POFs, such as easy manufacturing and high flexibility [24,25].

In our recent works, we have developed POF-based SPR sensors in different configurations, coupled with either biological (antibodies, aptamers, etc) [26] or chemical receptors such as molecularly imprinted polymers (MIPs) [27] for environmental [28], industrial and medical applications [29]. In particular, to obtain a general-purpose plasmonic sensor, suitable for different kinds of receptor layers (i.e., bio-receptors or MIPs), several years ago we designed and realized an SPR sensor in D-shaped POFs [30]. The Authors’ aim was to produce a very highly sensitive, robust, low-cost, and reliable SPR-POF sensor with the following specifications: a planar sensing area to spin the pre-polymeric mixture of MIPs and to drop the aqueous solutions; an SPR sensor with a wide refractive index range to work in different matrices; a sensor that could be used with a very simple and low-cost experimental setup connected to the Internet [31]; an SPR optical fiber sensor, with remote sensing capability, based on a highly flexible, durable, easy to manufacture and low cost device. To obtain these technical specifications we designed the SPR sensor as a 10 mm long D-shaped POF that can be monitored exploiting only two components: a white light source and a spectrometer.

**Figure 1:**
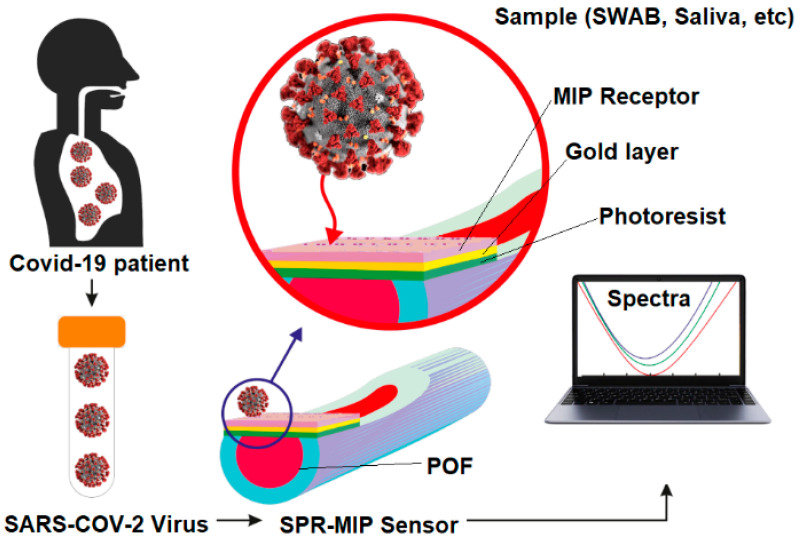
Outline of the Sensor for SARS-CoV-2 detection in different matrices.

In response to the current need for simple and fast methods to detect SARS-CoV-2, we designed and realized a first prototype of SPR-POF-MIP sensor for direct determination of SARS-CoV-2 virions in aqueous solutions. In particular, as shown in Figure 1, an SPR-POF sensor based on the configuration previously reported [30] was coupled with a novel kind of molecularly imprinted polymer nano-layer, properly designed for the specific recognition of the S1 subunit of SARS-CoV-2 spike protein. Figure 1 shows an outline of the proposed sensing approach for the SARS-CoV-2 detection in water solutions.

**Figure 2:**
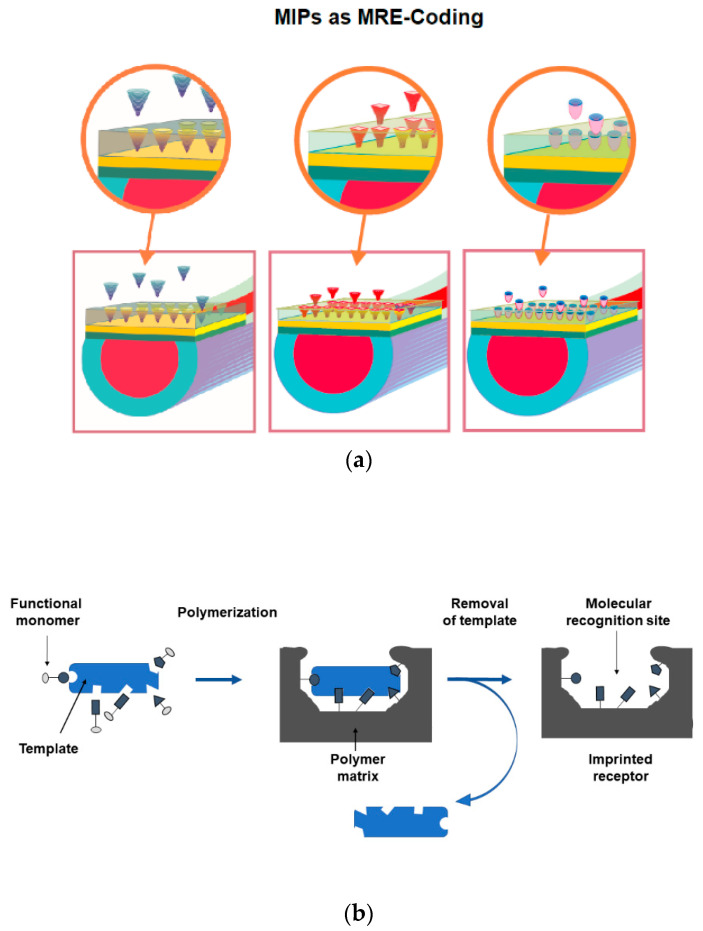
(**a**) Scheme of a general-purpose SPR-POF platform combined with different MIPs, as a versatile approach for optical-chemical sensing. (**b**) Outline relative to MIP’s realization process.

As the MRE is concerned, it is important to recall that the molecular imprinting technique can be used to create synthetic receptors for a specific analyte (biomimetic receptors) and these can be coupled with SPR-POF platforms to realize versatile, small size, low-cost optical-chemical sensors for different targets. As schematically shown in Figure 2, on the SPR-POF platform we could use different specific MIPs, imprinted for different substances, to realize a versatile sensing system, based on the same optical platform and an “MIP-coding”, as in the case of the personal computer, where hardware and software are present.

This diagnostic approach, based on a general-purpose sensor POF platform able to monitor “reprogrammable” MRE, will be highly desirable to face the next pandemic crisis.

In molecular imprinting techniques, the synthesis process involves a co-polymerization between appropriate functional monomers and a cross-linking agent, in the presence of the template molecule (the target analyte) [32,33]. For example, Figure 2a shows three different MIPs that interact with the target substances. The functional monomers coordinate the template molecule by interacting in very specific points, the cross-linking agent fixes the complex by creating a highly organized structure around it. At the end of the process the template is removed leaving the molecular recognition sites free. An outline on the MIP’s realization process has been reported in Figure 2b.

Synthetic receptors are very resistant, low-cost and able to work in wide ranges of pH and temperature [34,35,36]. Moreover, MIP receptors can be used by the optoelectronics industry to make optical-chemical sensors.

Presently we have no evidence of low-cost optical sensors based on molecularly imprinted polymers for the specific detection of SARS-CoV-2. We have evidence that MIPs are used (but not entirely described) as a potential synthetic MRE for SARS-CoV-2 in therapeutic applications [37] and as commercial synthetic receptors, see for instance the SARS-COV-2 nanoMIP for the SARS-CoV-2 spike protein developed by MIP Diagnostics (Colworth Park, Sharnbrook Bedford, UK). 

Moreover, an interesting MIP-based electrochemical sensor for the detection of SARS-CoV-2 nucleoprotein has been recently presented [38].

## 2. Materials and Methods

Reagents: Acrylamide (Aam) (CAS 79-06-1), N-*tert*-butylacrylamide (TBAm) (CAS 107-58-4), N,N’-methylenebisacrylamide (BIS) (CAS 110-26-9), 2-hydroxyethyl methacrylate (HEMA) (CAS 868-77-9), N,N,N’,N’-tetramethylethylenediamine (TEMED) (CAS 110-18-9), ammonium persulfate (APS) (CAS 7727-54-0), sodium dodecyl sulfate (SDS) (CAS 151-21-3), phosphate buffer solution 1.0 M were obtained from Sigma-Aldrich (Darmstadt, Germany) and used without any further purification. All other chemicals were of analytical reagent grade. The solvent was Milli-Q water.

The human serum albumin (BSA) (CAS 9048-46-8) and trypsin (CAS 9002-07-7) were from Sigma-Aldrich. The SARS-CoV-2 (2019-nCoV) Spike protein (S1 subunit, His-Tag) and MERS-CoV Spike protein (S1 subunit, His-Tag) were from Sino Biological (Düsseldorfer, Germany).

All optical measurements were performed using a very simple and low-cost equipment, including a halogen lamp at the input and a spectrometer at the output. In particular, the halogen lamp (HL-2000-LL, manufactured by Ocean Optics, Dunedin, FL, USA) used as white light source had an emission range from 360 nm to 1700 nm, whereas the spectrometer (FLAME-S-VIS-NIR-ES, manufactured by Ocean Optics) had a detection range from 350 nm to 1023 nm. The POF sensor was connected to the light source and to the spectrometer by two SMA connectors. The transmission spectra, along with data values, were displayed online on the computer screen and saved with the help of software provided by Ocean Optics, setting the integration time at 1000 µs and the averaging of the scans at 150. The SPR transmission spectra were normalized to a reference spectrum, achieved with air as surrounding medium, using the MatLab software (MathWorks, Natick, MA, USA).

## 3. Sensing Approach for Measuring SARS-CoV-2

### 3.1. Photonic Device Fabrication

To realize the SPR sensor, a D-shaped plastic optical fiber (POF) has been obtained by modifying a POF with a 980 µm core of polymethylmethacrylate (PMMA) and a 10 µm cladding of fluorinated polymer [30]. In particular, the cladding and part of the core were removed along half of the circumference by a polishing process that was carried out in two steps. First, we used a 5 μm polishing paper in order to remove the cladding and part of the core. Then, after about 20 complete strokes with an “8-shaped” pattern in order to completely expose the core, a 1 μm polishing paper was used for another 20 complete strokes performed according to an “8-shaped” pattern. The refractive index, in the visible range of interest, of the used POF is about 1.49 for PMMA (core) and 1.41 for fluorinated polymer (cladding). On the exposed POF core, about 0.1 mL of Microposit S1813 photoresist was deposited as a single drop and spun for 60 s at 6000 rpm. After 15 min in the oven at about 70 °C, a uniform layer of approximately 1.5 µm thickness was formed, with a refractive index (~1.61), higher than the one of the POF core. This layer was used to improve both the sensor’s performances and the adherence of the gold film. Finally, gold was sputtered by using a Bal-Tec SCD 500 machine to obtain a 60 nm thick film. In particular, to implement a low temperature sputtering process, it was repeated three times by applying a current of 60 mA, at 0.05 mbar of pressure, for 35 s (20 nm of gold per step). The gold film was preferred because it can be safely coated with bio-chemical/bio-mimetic receptors to realize specific sensors for several substances. The realized D-shaped sensing region was about 10 mm long and can be covered with a specific receptor to realize the biochemical sensor.

### 3.2. Receptor for SARS-CoV-2 Detection

We had to realize a very thin MIP receptor layer in order to optimize the sensing for the direct virion detection. In fact, the exponential decrease of the optical field from the metal-dielectric interface allows an effective interaction length that is no longer than a few hundred nanometers, so considering the virus size (falling in the range of one hundred nanometers, as well) the MRE thickness has to be kept as small as possible.

Since the specific interaction involves the molecular recognition of the S1 subunit and the anchoring of the entire virion, the polymer was grown on the transducer surface up to a thickness of less than 10 nm. This size allows to obtain enough superficial sites which are the only ones that can bind the spike proteins covering the external virion surface.

The resonance wavelength variation, due to the receptor deposition step, was used as an indirect measure of the receptors thickness, as reported in the Results and Discussion section.

The SPR optical fiber transducer has been suitably modified with an allyl thiol in order to allow to covalently bind the polymer layer to it. 

In particular, the gold surface of the transducer was modified by immersing it in a 10% *v/v* solution of allyl thiol in 80% *v/v* ethanol solution and 10% *v/v* water for 12 h. Subsequently, the platform was washed with Milli-Q water (flushing with 3 mL five times). Through this process a self-assembled monolayer with a terminal allyl group is formed.

After a treatment of the gold surface with an alcoholic solution of allyl mercaptan and the relative formation of a monolayer which exposes allyl groups, the polymerization reaction of the monomer mixture deposited on the SPR platform was initiated.

The polymeric receptor specific for SARS-CoV-2 was synthetized by using functional monomers able to interact, by non-covalent interactions, with functional groups of the S1 subunit of SARS-CoV-2 spike protein. The obtained complex was then frozen in the polymeric structure by a cross-linking reagent.

In order to meet the above requirements, we developed a novel strategy to realize a protein specific MIP receptor layer in non-denaturing conditions, as it has been described below. This molecular recognition strategy is object of a national pending patent (Moresense, Milan-Italy, Patent application number 102020000015145, filed on 24 June 2020). 

Acrylamide (Aam), N-t-butylacrylamide (TBAm), 2-hydroxyethyl methacrylate (HEMA) were added at 1:0.5:0.6 molar ratio, in 15 mM phosphate buffer (PB) pH 7.4. The final concentration of N,N′-methylene bisacrylamide (BIS) in the monomeric mix was 0.19M.

The pre-polymeric mixture was dispersed by sonication (sonic bath model VWR USC200T) for 10 min and bubbled with (nitrogen) N_2_ for 30 min at room temperature. The template (S1 subunit SARS-CoV-2 Spike protein) was added to the pre-polymeric mixture to the final concentration of 1 μM. Then APS (0.08% *w/v*) and TEMED (0.06% *w/v*) were added.

About 50 µL of the pre-polymeric mixture were dropped over the planar D-shaped sensing region and let polymerize for 15 min at room temperature, after which the reticulation process was stopped by washing the sensor surface with Milli-Q water. The template was removed by incubating trypsin 4.2 × 10^−8^ M for 2 h at room temperature on the sensor surface and then by washing with an SDS 5% (*w/v*) solution. The trypsin is commonly used to degrade proteins because of its proteolytic activity. 

The synthesis protocol conditions were preliminarily studied and optimized with an MIP specific for bovine serum albumin (BSA) protein, in which BSA was used as a test template molecule instead of SARS-CoV-2 spike protein.

The binding between the receptor and the target analyte, was tested on a sensor covered with a non-imprinted polymer (NIP) layer. In this case, the composition was the same as the MIP layer, previously described, but without adding any template. 

Finally, we have investigated the specificity of the imprinted receptor for SARS-CoV-2 by testing the cross-reactivity with the MERS-CoV spike protein. 

### 3.3. Experimental Protocol

All experiments were performed by dropping about 50 µL of the sample (spiked or real) over the planar sensing region of the SPR-POF sensor which was incubated at room temperature for ten minutes to let the interaction between the MIP sites and analyte occur. At the end of this incubation, a washing step with Milli-Q water was performed and subsequently the spectrum was recorded. By adopting this protocol, only the shift of the resonance wavelength determined by the specific analyte-receptor binding was measured, eliminating shifts due to bulk changes or non-specific interactions. 

We have repeated all the experimental measures five times to test the reproducibility of the developed sensor. For instance, to test the reproducibility of the measurements, from one anonymous negative and one anonymous positive patient, we have used five different swabs, usually utilized for the treatment/clinical assessment, to carry out these preliminary tests on negative and positive swabs. These swabs have been acquired in three consecutive days.

Before the analysis on real samples, a preliminary test was performed by incubating aqueous solutions of BSA and SARS-CoV-2 Spike S1 subunit at increasing concentrations.

Furthermore, a specificity test was carried out by incubating aqueous solutions of MERS-CoV Spike S1 and SARS-CoV-2 Spike S1 subunit at high concentration (1 µM).

Subsequently real swab samples in both universal transport medium (UTM) and physiological solution were tested. Serial dilutions performed with physiological solution were prepared for each sample and a dose-response curve was obtained by incubating from the most diluted to the whole sample.

### 3.4. RT-PCR Analysis

Nasopharyngeal (NP) swabs were collected from a patient, previously diagnosed as Covid-19 positive, for analysis by Operative Unit of Chemical-Clinical and Microbiological Analysis (San Luca Hospital, Usl Toscana Nord Ovest, Lucca, Italy), which is approved by the Ministry of Health in 2020 to test for SARS-CoV-2 infections. The laboratory employs the SARS-CoV-2 detection kits by SeeGene (Seegene, Seoul, Korea) for both viral nucleic acid extraction and RT-PCR-based amplification for 3 SARS-CoV-19 genes (STARMag and 2019-nCoV Assay kits, respectively, carried out in the Nimbus robot). The Allplex 2019-nCoV assay was designed so as to amplify three viral targets: the E gene (specific of the subgenus Sarbecovirus), the N and the RdRP genes (both specifics of the SARS-CoV-2). Regarding nucleic acid amplification, the Seegene assay was performed on the dedicated Bio-Rad CFX96 real-time thermal cycler according to the manufacturer’s’ instructions. The assay is approved for in vitro diagnostic use.

The first assessment of its performances by the manufacturer demonstrates a specificity of 100% and a limit of detection of 100 RNA copies/PCR reactions. NP swab samples were analyzed according to manufacturer’s instructions. The NP swab samples were also diluted 1:2 and 1:10 both in UTM and water respectively. All the dilutions were tested on both platforms starting from the same sample dilution.

## 4. Results

### 4.1. Preliminary Analysis: Spike Protein SARS-CoV-2 Detection

Our proposed device is based on the use of an universal plasmonic plastic optical fiber platform combined with a novel MIP receptor nano-film, designed for the specific recognition of the S1 subunit SARS-CoV-2 spike protein, for on-site specific and fast measurement of SARS-CoV-2. The preliminary development of the synthesis procedures and the characterization of the optical-chemical sensor were carried out using BSA protein as a test template molecule, to optimize the concentration of the initiators and the growth times of the surface layer. In fact, the BSA protein is cheaper than the commercial S1 subunit of SARS-CoV-2 spike protein.

**Figure 3:**
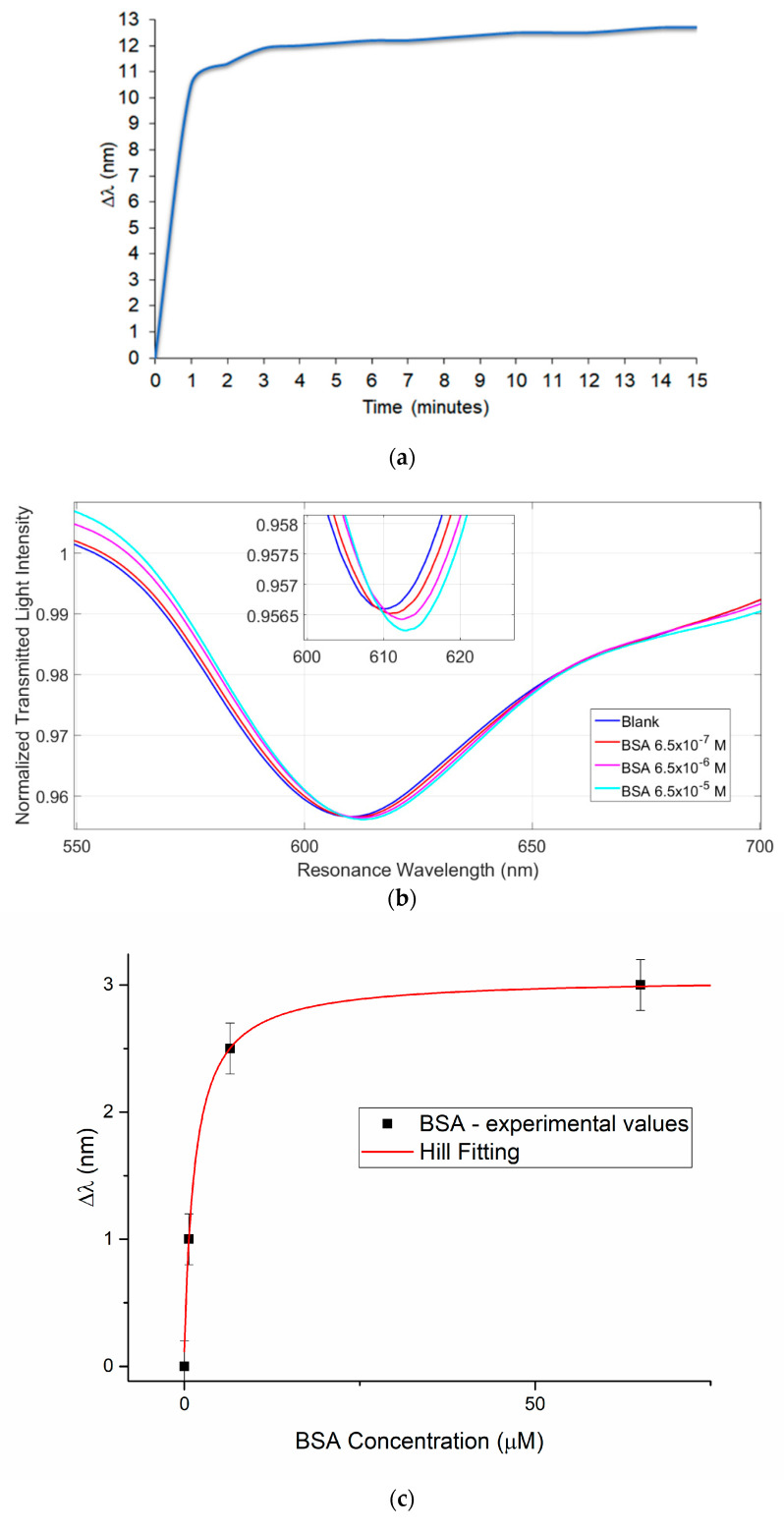

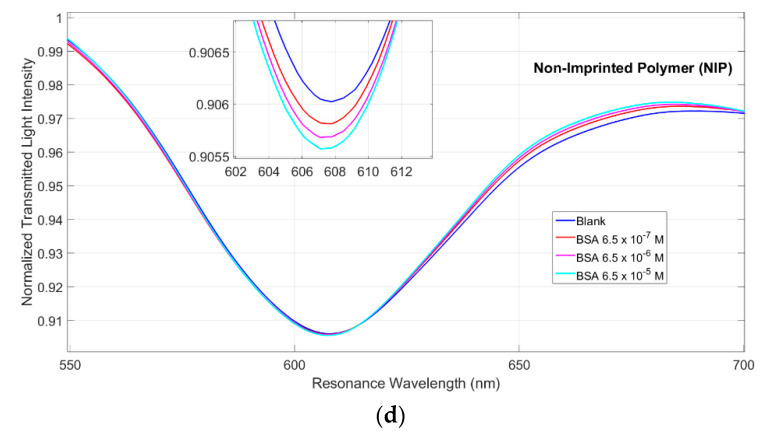
(**a**) Growth profile of MIP layer on SPR transductor registered with SPR Shift response. (**b**) SPR curves of different BSA protein concentrations in phosphate buffer pH 7.4 exploiting SPR-POF sensor combined with a specific MIP for Bovine serum albumin (BSA) protein. (**c**) BSA dose-response curve with Hill fitting of data. (**d**) SPR curves obtained with non-imprinted polymer (NIP) modified SPR-POF sensor at different protein concentrations in phosphate buffer pH 7.4.

The immobilization of the bio-chemical receptor on the optical sensor surface can be confirmed by the SPR results. In fact, before and after the functionalization step, the SPR spectra show a red-shift (an increase of the resonance wavelength) in presence of the same bulk refractive index (water, 1.332 RIU). As already demonstrated in [26,29], this shift, due to an increase of the refractive index of the medium in contact with the gold surface, indicates that the receptors were properly immobilized on the gold surface itself; so, in SPR platforms, when the thickness of the MIP layer on the gold surface increases, the average refractive index “seen” by the plasmonic phenomenon increases as well, and the resonance wavelength shifts to the right (red-shift). For example, a typical resonance shift, associated to a self-assembled monolayer (SAM) of receptors on an SPR-POF platform ranges from 5 nm to 20 nm, as previously found in the case of aptamers SAM, antibodies or chemical receptors [26]. In these cases, the SPR spectra have been used to check the functionalization process, whereas in this work the SPR spectra can be exploited to monitor the thickness of the MIP film in real time, allowing to stop its growth at the desired thickness value. In Figure 3a the resonance shift Vs time of polymerization reaction, is reported. After the growth of the MIP layer and the release of the template molecule from the sites, it was also possible to use the SPR technique to develop measurement protocols on the sensor to test BSA binding. In Figure 3b, different SPR curves obtained at different BSA concentrations in the phosphate buffer, are shown. When the protein is recognized by the receptor, the refractive index increases, and we observe a red-shift. Figure 3c reports the dose-response curve carried out for the BSA binding test, together with the Hill fitting of the experimental values. Table 1 reports the Hill fitting equation (when n = 1 it is similar to the Langmuir fitting) with its parameters used to calculate the chemical parameters of interest shown in Table 2.

In parallel, an NIP layer was synthesized and deposited on the sensor platform in order to check the imprinting process and to investigate the binding interaction with the target analyte. Binding tests were performed with this sensor and no Red-Shift was detected at different BSA concentrations, as reported in Figure 3d.

**Figure 4:**
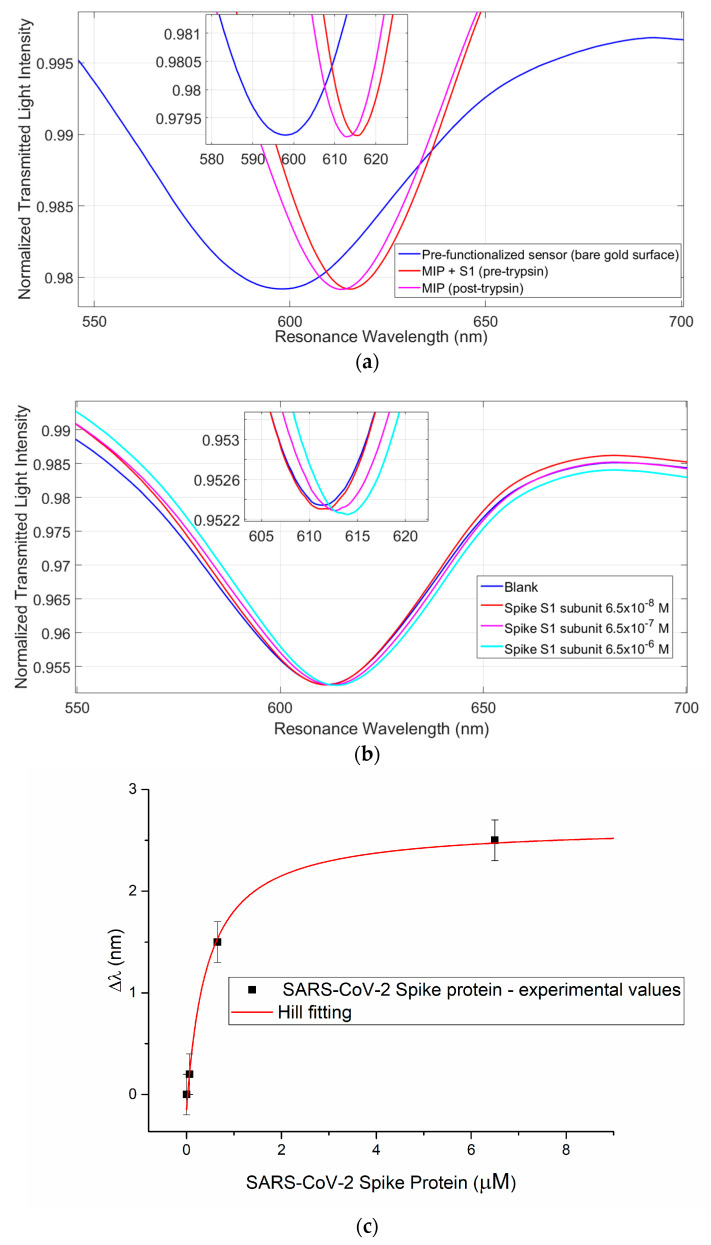

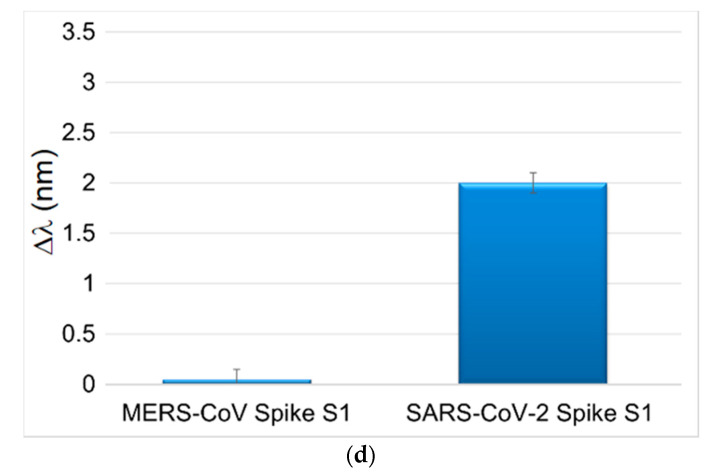
(**a**) Blue resonance peak: bare gold surface before the functionalization. Red resonance peak: red shifted resonance due to the MIP layer before template extraction with trypsin enzyme 4.2 × 10^−8^ M in buffer phosphate pH 7.4 and sodium dodecyl sulfate (SDS) 5%. Magenta resonance peak: Blue shift due to the freeing of sites from the template protein. (**b**) Response curves of Sars-Cov-2 Spike S1 subunit-MIP at different concentrations of protein. (**c**) SARS-CoV-2 Spike protein dose-response curve with the Hill fitting of the data. (**d**) Specificity test: sensor’s responses for MERS-CoV Spike protein and SARS-CoV-2 Spike protein, both with a concentration of 1 µM in UTM buffer.

The next step concerned the transfer of the collected information to the synthesis of the MIP for the specific S1 subunit SARS-CoV-2 Spike protein, using the commercial protein. Figure 4a illustrates the curves relative to the bare gold surface and the MIP modified platform before and after the extraction of the template protein. Once the S1 subunit MIP sensor was obtained, the spectra (useful to dose/response curve) were collected using commercial S1 subunit solutions (see Figure 4b).

Figure 4c reports the dose-response curve obtained for the SARS-CoV-2 Spike protein binding test together with the Hill fitting of the data. Table 3 reports the Hill fitting parameters used to calculate the chemical parameters of interest for SARS-CoV-2 Spike protein detection, reported in Table 4.

This MIP synthesized for the specific Spike S1 subunit SARS-CoV-2 protein is very specific for Spike SARS-CoV-2 protein in comparison to very similar substances, such as the Spike protein of MERS-CoV. Figure 4d shows the specificity test obtained by the same concentration of 1 µM of a MERS-CoV Spike protein and a SARS-CoV-2 Spike protein in UTM buffer. There is no detectable response of the developed sensor when a high concentration (1 µM) of MERS-CoV Spike protein is present, indicating a very good specificity of the MIP receptor layer.

From the chemical parameters reported in Table 2 and Table 4, the limit of detections (LODs) obtained for BSA and SARS-CoV-2 Spike protein detection, exploiting this type of MIP receptor combined with an SPR-POF probe, are lower than the LODs obtained by a similar SPR probe with different kinds of MIPs, i.e., in trinitrotoluene (TNT) detection by SPR-POF-MIP the LOD is equal to 51 µM [39]. The thin MIP film here presented could be the motivation of this performances’ improvement, together with the different molecular weight of the target.

### 4.2. SARS-CoV-2 Detection

**Figure 5:**
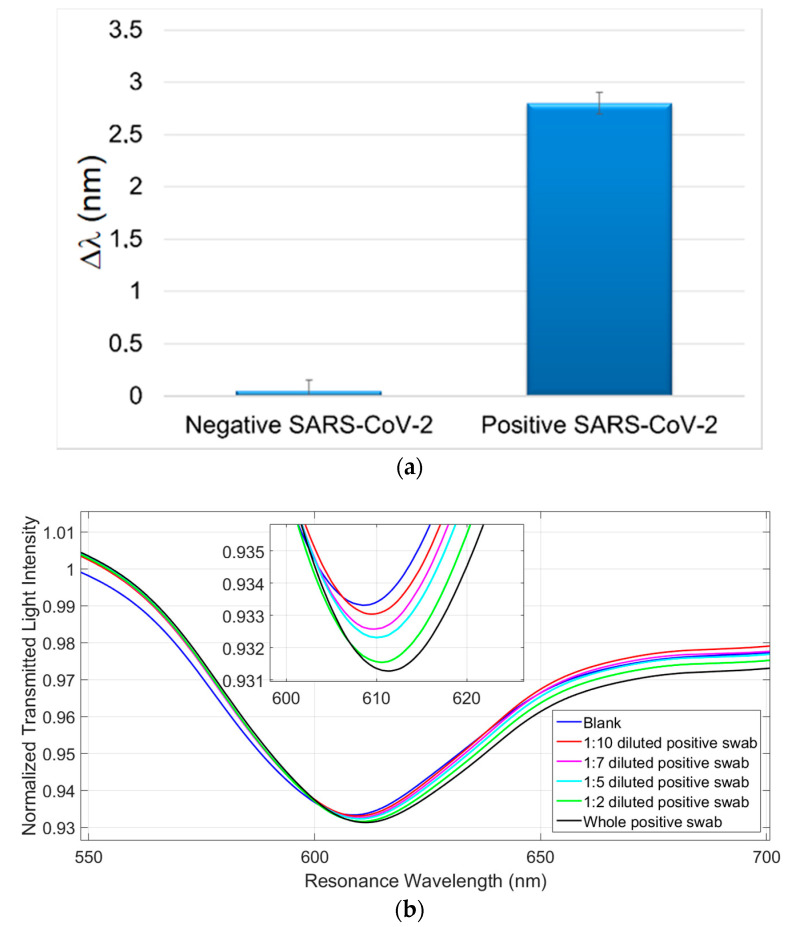
(**a**) Comparison of the response obtained by the MIP-SPR sensor with a negative SARS-CoV-2 swab in UTM (universal transport medium) and a positive SARS-CoV-2 swab in UTM (confirmed by RT-PCR). (**b**) Response curves of SARS-CoV-2 Positive UTM swab (36th RT-PCR cycle), at different dilutions, tested with SARS-CoV-2 MIP-sensor. The samples were diluted with physiological solution.

**Figure 6:**
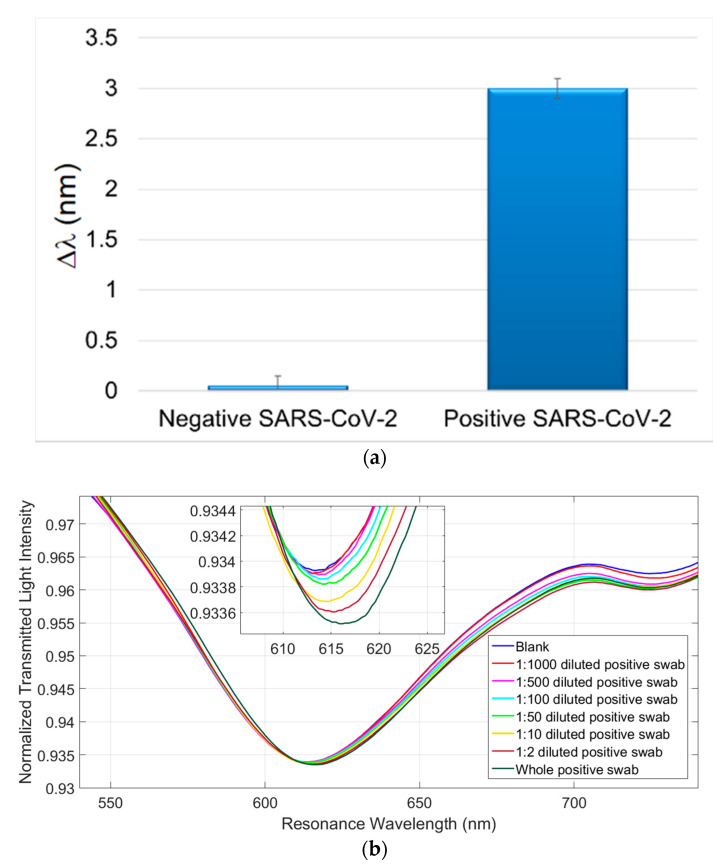
(**a**) Comparison of the response obtained by the MIP-SPR sensor with a negative SARS-CoV-2 swab (Physiological medium) and a positive SARS-CoV-2 swab (Physiological medium) (confirmed by RT-PCR). (**b**) Response curves of SARS-CoV-2 Positive swab (36th RT-PCR cycle) in physiological medium, at different dilutions, tested with SARS-CoV-2 MIP-sensor. The samples were diluted with physiological solution.

After the preliminary analysis on the detection of the SARS-CoV-2 Spike S1 subunit protein, we have tested the SARS-CoV-2 virions in two real matrices. Thus SARS-CoV-2 positive and negative samples in different matrices were tested. The samples were collected from a patient, previously diagnosed as Covid-19 positive, and analyzed in parallel with RT-PCR technique. In particular, Figure 5a and Figure 6a report the experimental results obtained by the SPR-POF-MIP sensor with real SARS-CoV-2 positive and negative samples of nasopharyngeal (NP) swabs in UTM and physiological solution (0.9% NaCl), respectively. Each experimental value is the average of five measurements and the error bars represent the upper bound of the standard deviation.

In Figure 5b and Figure 6b, the SPR curves of different dilution in physiological solution of NP swabs were reported, in order to investigate the detection sensitivity in both investigated media (UTM and physiological solution). For this NP swab, no resonance shifts in UTM are observed when the dilutions are higher than 1:10. The SARS-CoV-2 sensor sensitivity is higher in physiological solution, probably due to the complexity of the UTM formulation. Furthermore, it is important to stress that the SARS-CoV-2 measurement takes approximately 10 min.

The tests enabled us to appreciate SPR shifts on positive samples diluted up to 500 times (in physiological solution), thus allowing to detect very few viral units. The same diluted samples were tested in RT-PCR and the samples resulted positive only at 1:2 dilution. For instance, the whole positive swab in UTM shows positivity at gene E: 34,85; gene R: neg; gene N: 38,33; IC (internal control): 26,72 and the whole positive swab in physiological solution exhibits positivity at gene E: 32,39; gene R: 34,34; gene N: 34,89; IC: 26,65.

**Figure 7:**
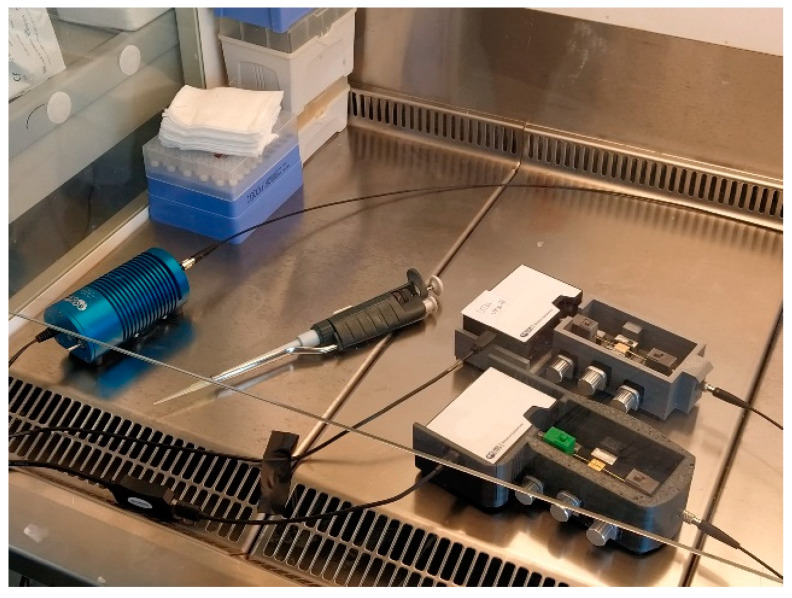
Picture of two SPR-POF-MIP sensor systems, used in parallel for the SARS-CoV-2 detection in real samples (UTM and physiological solution).

Figure 7 shows the picture of two sensor systems, used in parallel, for the SARS-CoV-2 detection in two different matrices (UTM and physiological solution).

## 5. Discussion

In a similar way to the graphene-based field-effect transistor (FET) biosensor for SARS-CoV-2, presented by Seo et al. [18], these preliminary results demonstrated that the proposed sensing approach could be used to develop a simple, low-cost, small-size, and rapid sensor device.

This novel SARS-CoV-2 sensor could be easily used on-site and connected to the Internet. Following the presented proof of concept, the subsequent experimental phase will be performed on a larger number of SARS-CoV-2 samples to achieve full validation of the approach. This will be carried out, also in collaboration with a company able to produce the sensing devices at industrial level, by comparing this SARS-CoV-2 sensor with the commonly recognized approach represented by RT-PCR.

In addition, the applied general-purpose plasmonic platform could be used to monitor a reprogrammable molecular recognition element which represents another highly desirable feature for the management of future pandemic crises. In fact, in consideration of the economic impact (or financial returns) of the device we are experimenting with ways of transforming our handcrafted product into one that can be industrially manufactured by exploiting, for example, silicon wafers. This should enable mass production, high quality, reliability and low-cost due to the advantages of the typical processes of the semiconductor industry. Furthermore, not only does a planar approach allow to produce single devices, but it also allows to devise complex arrays of a variety of sensors with the same or different geometries and functionalization to support fast screening tests for a large number of people. Moreover, the main strategic advantage of our approach is that the industrialized optical platform is the same for many different applications, which means that it could be used for the current pandemic and for future ones as well.

Proper attention should just be paid to the appropriate design of the MIP on top of it that, being the last step of the sensor production process, allows to implement a sort of specific MIP coding, creating a really “open” and extremely versatile sensing platform.

## 6. Conclusions

The proof of concept of SARS-CoV-2 selective detection has been demonstrated exploiting a low-cost optical-chemical sensor system. The refractive index of an MIP nanofilm, deposited on the sensing gold film, changes when the binding with the SARS-CoV-2 virion occurs. The MIP’s refractive index variation has been monitored by a simple plasmonic D-shaped POF-platform. These preliminary results have proven an elegant approach to achieve fast SARS-CoV-2 detection at the point-of-care. The reported data demonstrate a prototype sensor capable of detecting the spike protein of SARS-CoV-2 in various solutions and the virions as well. Nevertheless, the actual ability of the technique to be exploited in the detection of a SARS-CoV-2 infection requires further validation on a large number of SARS-CoV-2 samples and the thorough comparison with RT-PCR, exploiting a sensor system realizable at an industrial level.

## Figures and Tables

**Table 1:** Hill parameters of bovine serum albumin (BSA) detection.

Hill Equation: ∆λ_c_ = ∆λ_0_ + (∆λ_max_ − ∆λ_0_) × (c^n^/(K^n^ + c^n^))								
Δλ_0_ (nm)		Δλ_max_ (nm)		K (µM)		n	Statistics	
Value	Stand. Err.	Value	Stand. Err.	Value	Stand. Err.	Value	Reduced Chi-Sqr	Adj. R-Square
0.115	0.360	3.055	0.068	1.510	0.566	1	0.170	0.996

**Table 2:** Chemical parameters for bovine serum albumin (BSA) detection.

Value	BSA-Parameters
1.947 (nm/µM)	Sensitivity at low conc = (Δλmax − Δλ_0_)/K Low conc Hypothesis: (c << K) Hill Equation at low conc (with n = 1): Δλ_c_ ≈ [(Δλmax − Δλ_0_)/K] × c
0.37 (µM)	Limit of detection (LOD) LOD = [(2 × standard deviation of blank) / Sensitivity at low conc]
0.662 (µM)^−1^	K aff = 1/K

**Table 3:** Hill parameters of SARS-CoV-2 Spike protein detection.

Hill Equation: ∆λ_c_ = ∆λ_0_ + (∆λ_max_ − ∆λ_0_) × ( c^n^/(K^n^ + c^n^))								
Δλ_0_ (nm)		Δλ_max_ (nm)		K (µM)		n	Statistics	
Value	Stand. Err.	Value	Stand. Err.	Value	Stand. Err.	Value	Reduced Chi-Sqr	Adj. R-Square
−0.149	0.188	2.647	0.102	0.431	0.123	1	0.317	0.991

**Table 4:** Chemical parameters for SARS-CoV-2 Spike protein detection.

Value	SARS-CoV-2 Spike Protein-Parameters
6.483 (nm/µM)	Sensitivity at low conc = (Δλmax−Δλ_0_)/K Low conc Hypothesis: (c << K) Hill Equation at low conc (with n=1): Δλ_c_ ≈ [(Δλmax − Δλ_0_)/ K] × c
0.058 (µM)	Limit of detection (LOD) LOD = [(2 × standard deviation of blank) / Sensitivity at low conc]
2.318 (µM)^−1^	K_aff_ = 1/K
